# Provings and Clinical Observations with High Potencies

**Published:** 1891-12

**Authors:** Malcolm Macfarlan

**Affiliations:** Philadelphia


### PROVINGS AND CLINICAL OBSERVATIONS WITH HIGH POTENCIES.

Malcolm Macfarlan, M. D., Philadelphia.

When a medical officer in the United States service, stationed in Southern Alabama in the summer of 1865, I was frequently baffled in my efforts, as were others, to cure dysentery, so common and fatal among the troops. I wrote North to a young friend, not then a physician, who sent me Hahnemann's *Organon*, *Hull's Jahr*, and, later on, Fincke's book, and some of his and other potentized medicines. I read the books and made successful use of the remedies, particularly Corrosive Sublimate and Opium. Next year I began systematic provings with the medicines sent, and others of my own preparation. My plan was then, and has been ever since, to work with one remedy for several weeks, giving it to an individual in water every hour or so until symptoms developed, as many persons taking it at the same time as I could conveniently obtain. While with some I could get no satisfactory results, yet out of a sufficient number of others who were sensitive I observed symptoms common to all those affected to enable me to understand the remedy, and, most important of all, to convince me beyond a doubt of the truths of Hahnemann's *Organon*. Just as there are some who will not be affected by causes which produce disease in others, so a potentized medicine will not always bring out its characteristic symptoms on some provers, although my observation teaches me it is much more likely to act, or is more to be depended on than infection or similar cause of disease. Idiosyncrasies are called out in proving, peculiar to an individual, of which I have taken no notice. They may be produced often by other medicines, and so are misleading. My object has been to collate a *few* reliable symptoms, not as *many* as possible. What is written is, to the best of my knowledge, true, and was worked out originally for my own guidance and information. It is now given for what it may be worth to the profession. There is nothing herein copied nor obtained from any one else. The symptoms are responses of nature as far as I could understand her. From time to time I have given these records, in a fragmentary way, to others, but for the first time do they appear in this completed form. The work was apparently so small and imperfect, so slow and difficult to compile that I have long hesitated to publish these results of my observations for a period of twenty-five years. It is given as an encouragement to those traveling the same road; perhaps as a help, and especially to those investigating for themselves without accepting the dictum of anybody. I have also looked upon it as a contribution and an acknowledgment for the benefit received from the teachings of others. The provings, as far as possible, are in the language of those on whom they were made.

Just now, when a strong tide is setting against anything like *Hahnemannian* Homœopathy, and its practice dwindling and almost obsolete, it may induce others to investigate or prove highly potentized remedies, because it is believed by those who use them that they are curative in cases where other remedies fail. It is not possible to have any belief in them otherwise. He who ridicules without having made a patient investigation is not competent to pass an opinion on their merits. I am sure the result will be surprising, and make a convert of any candid investigator. In this way can the losing cause be placed on a sure basis. The methods and brilliant cures of the former generation of homœopaths, by whom the system was established, are almost unknown, and to-day there is practically no difference between the allopath, who uses parvules and triturates, and the homœopath usually met with.

The remarks are mostly made concerning Fincke's high potencies, but are equally true of those made by Jenichen and others, as I have frequently verified.

The medicines were given in water as a rule. *Patients never knew that they were making provings of medicines.* The pellets were put in one-half a tumbler of water and a teaspoonful at least every two hours, often every hour. Symptoms generally occurred on the third day. The provers in many cases had local ailments, fractures, injuries, etc., which did not interfere much with their general health or complicate medicinal symptoms.

### Aconite-nap.$^{50M}$.

Caused such free sweating, to make use of patient's language, you could have wrung out his shirt. It checked or modified night-sweats for awhile in a number of consumptives, and moved the bowels three times a day, liquid stools. Almost the first effect of highly potentized Aconite is to cause perspiration quickly.

Profuse sweat during sleep. Great inconsolable anxiety. Anxious feeling. Rash like measles, lasting only a day or so. Free sweat, with pain in joints and muscles.

### Actæ-racemosa$^{5C}$.

Given to a man every hour during the day for two weeks caused slightly bloody urine; urination frequent. Sick feeling in epigastrium, costive.

It was noticed that it suppressed menstruation in a certain number of females, in addition to urinary symptoms.

### Adeps-suis.$^{1M}$ (F.).

*Constipation.*—Hard, dry stool, cramps in stomach.

Bowels that were constipated now move daily, weakness after the stool, cramps about the navel, severe pains between the last lumbar vertebra and sacrum.

Such great weakness behind her knees in popliteal space that the prover could hardly get up. Hips and elbows painful on motion.

Hard, dry, insufficient stool not easily expelled. Caused bowels that were constipated to move daily. I have frequently verified this in curing constipation when there was loss of expulsive power in rectum. Stools dark, hard, and dry.

### Æsculus$^{50M}$.

Headache, drowsy, walking difficult, so weak; legs ache; all provers complained of extremities being much affected, bowels loose.

Hands and face swell up enormously and increased so after washing. Pimples appeared on face and body. This was noticed in only one case where medicine had been given for several weeks.

Many pimples on the face, few on the body, in most provers.

### Agaricus-musc.$^{47M}$.

Completely relieved a severe bearing-down sensation in the uterus, which I had been treating unsuccessfully off and on for a year and a half. After some weeks it returned slightly, when she had not been taking the remedy, but was cured by a second exhibition of it. Soreness in ovaries.

Given to a number of females, produced general stiffness. Arms and back of neck stiff. Little hard, red pimples scattered all over the body, like flea bites. Never has been so sleepy as during this last week. (Second week of the proving.)

Beating on *top of head* toward forehead, cured bearing-down pain, *very thirsty,* never has been so thirsty, twisting pain in umbilicus, pain in both hip-joints, gets up stiff like an old rheumatic, stiff joints in general. *Soreness* deep in two spots four inches on either side of middle of sternum, hurts or weakens her to breathe deeply or speak continuously, little hard pimples break out on side of her nose and about her lips.

Given every hour for a week caused cramps and chills as if she were going to have the ague, couldn't remain up, had to lie down, had to pass water all the time, severe bearing-down pain when she attempted to walk. Talking in a loud voice and deep breathing hurt her in the right ovarian region, bowels now move twice a day instead of once. These symptoms were most violent on the third day.

### Agaric-mus.$^{2M}$.

Sick at stomach, threw up a good deal, abdomen very sore, below the navel sore to touch.

### Ailanthus$^{45M}$.

September 20th, 1872.—Mary W., widow, aged forty, medicine every two hours during the day, symptoms after a week: Had a slight trouble with her knee as if sprained ; had always been in good health. " If she touches or presses her lip it will puff up and appear to be sore, there is now a ragged, deep little sore near the angle of the mouth ; the lower lip has a crop of angry blisters about it. Violent itching sensation about the ears, back of the neck and less in degree about the face. The skin has divided off into red raised blotches ; intense desire to scratch the blotches. A water blister came on the end of her thumb ; small blisters appeared at tips of fingers ; her chin one day was bright scarlet ; another day her ear had the appearance of erysipelas, from slight rubbing. Skin of face very itchy and becomes very red on the least rubbing."

Curative in a case of chronic acne in a young girl.

### ALCOHOL-SULPH.$^{50M}$.

Pains nearly every day, mostly after dinner, in head, through temples, very sharp ; numb all through top of her head, in scalp.

### ALETRIS-FARINOSA$^{45M}$.

*Severe pains* in the rectum or anus, frequent desire to have a stool ; diarrhœa on second, third, and fourth day ; the pain when she had a movement was violent, felt as if she was forcing a passage through an obstruction ; feeling of exhaustion ; when she stoops down head gets dizzy, eyes feel sore and dim ; spits a good deal and raises mucus ; feels as if she wanted to cough and cannot.

### ALUMINA$^{91M}$.

*Can pass urine only during stool.* Frequent stools. Stools with traces of blood.

### AMMON-CARB.$^{5C}$.

Given for ten days every two hours, produced a red rash like erythema with a great deal of heat ; burning and fever ; burning feeling in eruption ; skin raised in welts in some places.

Dry cough at three A. M., or toward morning, occurred in a great number of provers.

### ANTIMONIUM-CRUD.$^{60M}$.

Eyelids inflamed, water a great deal, can hardly keep them open ; feels sleepy ; eyes feel as if too heavy to keep open, very sore ; worse in morning ; left eye most inflamed.

### APIS-MELL.$^{85M}$.

Loose bowels; watery, green, slimy stools; no vomiting, etc. Repeated verifications of this. Cured with this remedy many cases of cholera infantum. Frequent green stools with disposition to congestion of brain; starting, jerking, eyes rolling; later on disposition to constipation.

Caused great tearfulness or disposition thereto, verified many times.

Quickly checked shrill screaming in a severe case of congestion of brain, young child teething.

*Cured* a very bad case, over a year's standing of chronic diarrhœa with many small passages of blood and mucus in a woman at critical period. Curative in difficulty of urination common to children. Often relieved very red enlarged tonsils. Scalp sensitive in many provers.

### APIUM$^{69M}$.

Dull aching in forehead; severe pains in bowels; sharp pains in ear when chewing or moving jaws; eyes feel as if sand in them. Some provers vomited while taking the remedy.

### APOCYNUM-CANN.$^{60M}$.

Feels as if she was hungry; and when she tries to eat, food appears to settle in epigastrium, becomes sour; continued distress in epigastrium, feels as if she could do nothing but cry; does not want to speak, very low-spirited, weeping; tongue greatly coated, brownish white; dizzy, headache, drowsy in afternoon, restless and wakeful at night. Urine pale and greatly increased in quantity.

### APOCYNUM-CANN.$^{60M}$.

Patient becomes very drowsy and vomits very often; pulse is very slow. Cured a most inveterate case of wetting the bed at night in a girl aged twenty, affected all her life, and treated by many without success.

Cured general stiffness of legs and body, painful on motion.

Cured frontal headache, sick at stomach, restless at night. *Stiff knees* often verified this symptom—stiffness not similar to rheumatic trouble.

### ARANEA-DIADEMA$^{45M}$.

Male prover. Produced a boil on left side of penis, near pubes; constant desire to pass water, but with difficulty. Severe pain along the urethra, extending from the glans.

Woman, misty sensation before her eyes; felt so tired that it appears as if she would drop; bowels now move daily, which were four or five days constipated. Vivid dreams, screams out, and cannot sleep again.

After taking the remedy two weeks, had to stop it, as it partially suppressed his urine. He passed only four or five ounces during the day; no burning. Urine appeared darker than natural; had to stand and wait a long while before it would come. Sensitive to pressure on either side of his bladder; no energy. Urinary symptoms verified in other provers, showing diminished secretion.

### ARGENTUM-NIT.$^{45M}$.

In a woman produced severe symptoms, similar to angina pectoris, difficulty of breathing, choking, and sensation of pain about the heart; her friends thought she would die; sent for me in a great hurry. Attacks lasted fifteen or twenty minutes. In others produced distressed spasmodic breathing.

### ARGENT-NIT.$^{45M}$.

Swollen left side of the face, with a great deal of heat and burning; lips greatly swollen, inside of the mouth much swollen; earache, marked soreness in flesh and limbs. Female provers.

### ARNICA-MONTANA$^{2C}$.

Every hour during the day, for two weeks, produced a rash like scarlatina at first, then papular eruption; which changed in a few days to a crop of small boils, half the size of a pea, all over the body. One prover.

### ARSENICUM-ALBUM$^{103M}$.

Sick at stomach; did not vomit; great weakness, dizzy headache on top of head, no appetite, no particular thirst, slight fever and sweat every afternoon, some difficulty in breathing, like asthma. She was compelled to lie down, she was so weak; her left ear discharges slight moisture; face swollen up, sighing for breath, panting on exertion; bowels have been regular; watery discharge from nose, *fainted* several times during the week.

### ARSENICUM-ALBUM$^{6M}$.

Appears to be most useful in ophthalmia when photophobia is present. I have often verified this symptom.

Fainted several times while walking out. She had never fainted before. Had been taking the medicine four days when this occurred. Cannot sleep after three A. M. She gets up in a fright from vivid dreams. Very low-spirited.

*Night-sweats,* chilly and feverish, mostly toward sundown. Throat quite sore and painful, back aches, buttocks sore to touch, appears as if a lump came like a hurt in her throat. It seems as if she was swollen throughout her whole body, pain extending from her head to her right shoulder and down her right side. Could not sleep because of anxiety. Fear that some evil will overtake her.

### ARSENIC-ALBUM.$^{6M}$.

Prover wakes in a horrid fright at three A. M. Cannot be convinced but that something dreadful will happen him.

Fainting in a woman when out walking verified often, never happened before.

Cured a case of ascites following chronic diarrhœa. Made comfortable many suffering from Bright's disease—with general dropsy.

Eyes misty at times, fainty feeling, could not see well at times, eyes not inflamed, did not water, feels as if she had a load in upper part of both lungs, feels as if she would smother.

Highly curative in some cases of scrofulous ophthalmia and ophthalmia tarsi where the disease is communicated, as in children.

Cured cases of scald head, belching, frequent and small stools, inability to sleep well because of mental anxiety. In giving Arsenic to those with ophthalmia it produces great intolerance of bright sunlight, not candle or gas light, cannot endure the rays of sun or bright daylight. Caused feeling of weakness as if he would faint.

[TO BE CONTINUED.]

